# Comparative Study of Multi-Delay Pseudo-Continuous Arterial Spin Labeling Perfusion MRI and CT Perfusion in Ischemic Stroke Disease

**DOI:** 10.3389/fninf.2021.719719

**Published:** 2021-08-11

**Authors:** Xi Xu, Zefeng Tan, Meng Fan, Mengjie Ma, Weimin Fang, Jianye Liang, Zeyu Xiao, Changzheng Shi, Liangping Luo

**Affiliations:** ^1^Medical Imaging Center, The First Affiliated Hospital of Jinan University, Guangzhou, China; ^2^Department of Neurology, The First Affiliated Hospital of Jinan University, Guangzhou, China; ^3^Department of Neurology, Shun De Hospital of Jinan University, Foshan, China; ^4^Department of Medical Imaging, Sun Yat-sen University Cancer Center, Guangzhou, China; ^5^Engineering Research Center of Medical Imaging Artificial Intelligence for Precision Diagnosis and Treatment, Guangzhou, China

**Keywords:** ischemic stroke, pseudo-continuous arterial spin labeling, CT perfusion, cerebral blood flow, arterial transit time, mean transit time

## Abstract

With the aging population, stroke has gradually become the leading cause of death and disability among adults. It is necessary to verify whether multi-delay pseudo-continuous arterial spin labeling (pCASL) MRI can be used as a standard neuroimaging protocol in the patients with ischemic stroke. We aimed to investigate the clinical utility of multi-delay pCASL for evaluating cerebral perfusion in ischemic stroke disease. Twenty-one ischemic stroke patients [18 men and 3 women; median age, 62 years (age range, 37–84 years)] were enrolled in this study. All patients underwent examinations, including the multi-delay pCASL protocol (using 6 PLDs between 1,000 and 3,500 ms) and computed tomography perfusion (CTP). The cerebral blood flow (CBF) and arterial transit time (ATT) maps were obtained by the multi-delay pCASL protocol, while CBF and mean transit time (MTT) maps were derived by CTP measurements. Based on the voxel level analysis, Pearson correlation coefficients were used to estimate the associations between the two modalities in the gray matter, white matter, and whole brain of each subject. Moderate to high positive associations between ASL-CBF and CTP-CBF were acquired by voxel-level-wise analysis in the gray matter, white matter, and whole brain of the enrolled patients (all *P* < 0.005), and the average Pearson correlation coefficients were 0.647, 0.585, and 0.646, respectively. Highly significant positive correlations between ASL-ATT and CTP-MTT were obtained by voxel-level-wise associations in the gray matter, white matter, and whole brain (all *P* < 0.005), and the average Pearson correlation coefficients were 0.787, 0.707, and 0.799, respectively. In addition, significant associations between ASL and CT perfusion were obtained in the gray, white matter and whole brain, according to the subgroup analyses of patient’s age and disease stage. There is a correlation between perfusion parameters from multi-delay pCASL and CT perfusion imaging in patients with ischemic stroke. Multi-delay pCASL is radiation-free and non-invasive, and could be an alternative method to CT scans for assessing perfusion in ischemic stroke disease.

## Introduction

The Global Burden of Disease Study in 2015 provided a comprehensive assessment of 249 all-cause and cause-specific deaths across 195 countries and regions from 1980 to 2015; stroke was determined to be the second-leading cause of death worldwide (Wang et al., 2016). The 2019 statistical report from the American Heart Association on heart disease and stroke revealed that approximately 795,000 people experience a new or recurrent stroke each year (Benjamin et al., 2019). With the aging of the population, stroke has gradually become a leading cause of death and disability among adults in China. The social burden of stroke has gradually increased because of the increased morbidity and mortality (Vilela and Rowley, 2017).

Stroke is a type of cerebrovascular disease characterized by the symptoms of cerebral ischemia or cerebral hemorrhage, and cerebral ischemia is the main cause of stroke (Benjamin et al., 2019). Early diagnosis and appropriate treatment of the disease can effectively improve the prognosis of patients. MRI and CT are routine imaging modalities that play important roles in evaluating the brain condition of patients. Because of the pathophysiological changes in stroke patients, the assessment of microcirculation and cerebral perfusion is of great value for diagnosis and treatment.

The development of CT image analysis to assess the function of different organs (e.g., brain, heart, carotid artery, etc.) constitutes a promising strategy for evaluating normal and abnormal physiology (Xu et al., 2018; Zhang et al., 2018). As known errors are associated with bolus-based perfusion measurements (Wintermark et al., 2005), CT perfusion (CTP) is not regarded as the gold standard for calculating hemodynamic parameters. However, it is routinely used in clinical practice for perfusion evaluation, because it provides relatively accurate hemodynamic parameters, such as cerebral blood flow (CBF) and mean transit time (MTT). It’s noting that the radiation damage and contrast medium may not be suitable for some individuals. Arterial spin labeling (ASL) perfusion MRI has gradually applied in the clinic without radiation damage. Previously studies have generally adopted a single post-labeling delay (PLD) time (Wang et al., 2014), typically between 1.5 and 2 s, which may underestimate perfusion due to prolonged arterial transit time (ATT) in ischemic stroke (MacIntosh et al., 2010). In recent years, multi-delay ASL sequences have been used to overcome this limitation. Wang et al. (2014) concluded the correlations between multi-delay pCASL and CTP in moyamoya disease, and indicated ASL could be a part of neuroimaging protocols in the moyamoya disease. They advised warranted studies of ischemic stroke, as the radiation-free and non-invasive ASL can provide perfusion information without the use of contrast agent. MacIntosh et al. (2010) adopted multi-delay pCASL (PLD times were set between 500 ms and 2,500 ms, a total of 9 intervals) in patients with acute ischemic stroke in a recent research. Wang et al. (2013) made a comparison between multi-delay ASL perfusion MRI and dynamic susceptibility contrast (DSC) enhanced perfusion imaging in acute ischemic stroke disease. The results showed highly correlations between pCASL and DSC CBF measurements. In our study, a wider range of PLD times was adopted, totally 6 PLD times between 1,000 and 3,500 ms were used to investigate the accuracy of multi-delay pCASL perfusion MRI for estimating cerebral perfusion in ischemic stroke patients, using CTP as the reference standard. In this study, we aimed to explore the feasibility and clinical utility of multi-delay pCASL by comparison with CTP imaging in ischemic stroke.

## Materials and Methods

### Patients

**FIGURE 1:**
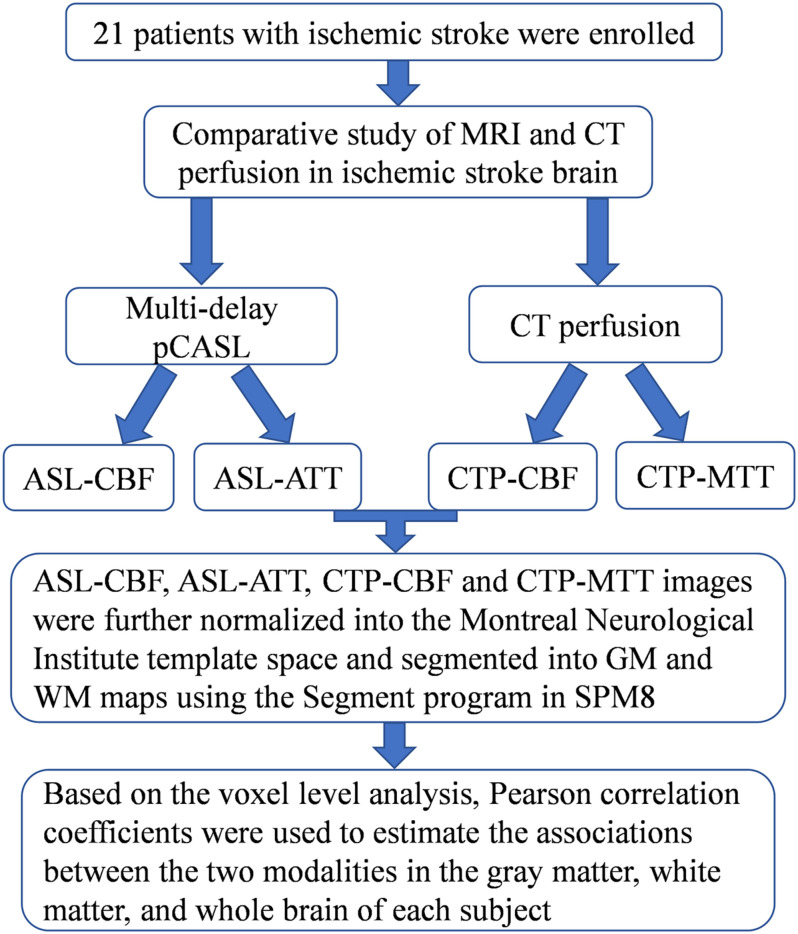
Flowchart of the comparison between ASL and CT perfusion.

The study was conducted in accordance with the principles of the Declaration of Helsinki, and the study protocol was approved by the institutional review board of our hospital. A total of 21 patients with ischemic stroke [18 men and 3 women; median age, 62 years (age range, 37–84 years)] were enrolled between June 2017 and March 2018. All patients underwent multi-delay pCASL and CTP examinations (Figure 1).

The inclusion criteria were as follows: (1) All patients were confirmed as ischemic stroke according to clinical symptoms and imaging diagnosis (MRI or CT images); (2) To reduce additional CT-associated radiation damage, patients who required CTP examination based on their condition were enrolled in the study; (3) Both CTP and ASL examinations were performed within 24 h.

The exclusion criteria were as follows: (1) Patients complicated with severe parenchymal organ disease (heart, lung, liver and kidney); (2) Patients complicated with brain trauma, brain tumor, intracranial hemorrhages, craniocerebral infection and other mixed factors; (3) Patients with a cardiac pacemaker, non-titanium alloy stent or internal plate fixation *in vivo*; (4) Unable to complete the examination due to claustrophobia.

### Image Acquisition

All included patients underwent CTP on a Toshiba Aquilion One scanner, which was set at 112∼187 mA and 80 kV. And the minimum section thickness was 0.5 mm. CTP scan was initiated after injection of non-ionic iodinated contrast agent (Ultravist, 370 mg I/L; 50 ml at a rate of 6 ml/s) and physiological saline (30 ml at rate of 6 ml/s), using a power injector. The dynamic volume scan mode was turned on immediately after a delay of 7 s following the intravenous injection of contrast agent. The first period was used as a mask; 11–36 s was used for the arterial phase, and continuous scanning was performed at intervals of 2 s; 40–60 s was used for the venous phase for continuous scanning at intervals of 5 s. In total, 19 image acquisitions were performed with a total scan time of 60 s.

All enrolled patients underwent MRI on a GE Discovery MR750 3.0T System, using an 8-channel phased array head coil. The MRI protocol included T1-weighted imaging (T1WI), T2-weighted imaging (T2WI), fluid attenuated inversion recovery (FLAIR), diffusion weighted imaging (DWI), and multi-delay pCASL. For multi-delay pCASL, the imaging parameters were as follows: the delay time was between 1,000 and 3,500 ms, for a total of 6 PLDs. Filp angle (FA) = 111°, repetition time (TR) = 5,436 ms, echo time (TE) = 24.2 ms, slice thickness = 5 mm, spacing between slices = 0 mm, FOV = 220 mm × 220 mm, acquisition matrix = 550 × 6, number of excitations (NEX) = 1.

### Processing of ASL and CTP

The post-processing of CTP data was performed on Vitrea Fx 6.3 image post-processing workstation by a senior radiologist. CTP-CBF and CTP-MTT perfusion maps derived from CT perfusion images using delay-insensitive blockcirculant singular-value decomposition (bSVD) post-processing method referring to existing described procedures (Wintermark et al., 2001).

Mean perfusion difference images were generated for each PLD. Both ASL-ATT and ASL-CBF perfusion maps were computed online. The ASL-ATT map was converted using the weighted delay method as previously described (Dai et al., 2012; Wang et al., 2014).

Pre-processing was performed using Data Processing and Analysis of Brain Imaging (DPABI_V2.3)^1^, which is based on Statistical Parametric Mapping (SPM8)^2^. DPABI was developed in MATLAB 2013 (The MathWorks Inc., Natick, MA, United States). ASL-CBF, ASL-ATT, CTP-CBF, and CTP-MTT images were further normalized into the Montreal Neurological Institute template space using SPM8. Based on the registered 3D T1W images, the gray matter (GM) and white matter (WM) masks were extracted. ASL-CBF, ASL-ATT, CTP-CBF, and CTP-MTT images were segmented into GM and WM maps using the Segment program in SPM8.

### Statistical Analysis

Voxel-wise analysis of the gray matter, white matter, and whole brain was conducted by DPABI: Pearson correlation coefficients were calculated across voxels between the two modalities in the gray matter, white matter and whole brain of each subject as previously described (Wang et al., 2014). Subgroup analyses based on age of patient and disease stage were performed to study the associations between ASL and CT perfusion.

## Results

### Clinical Characteristics of Enrolled Patients

In total, 21 patients [18 men and 3 women; median age, 62 years (age range, 37–84 years)] with confirmed ischemic stroke were enrolled in this study. Of 21 patients, fifteen (71.4%) had hypertension, 3 (14.3%) patients had diabetes, and 10 (47.6%) patients had elevated triglyceride levels (Table 1).

**TABLE 1:** clinical characteristics of patients at admission (*n* = 21).

**Variable**	**Patients (*n* = 21)**
**Patient demographics**	
Median age, years (range)	62 (37–84)
Male (n,%)	18 (86)
Female (n,%)	3 (14)
Disease stage	
Acute stage (n,%)	5 (24)
Subacute stage (n,%)	8 (38)
Chronic stage (n,%)	8 (38)
**Laboratory test**	
Blood pressure, mmHg	
Median systolic pressure (range)	143 (111–160)
Median diastolic pressure (range)	80 (68–107)
Hypertension (n,%)	15 (71)
Median fasting glucose, mmol/L (range)	4.97 (3.96–9.49)
Diabetes (n,%)	3 (14)
Median triglyceride, mmol/L (range)	1.64 (0.64–4.85)
Elevated triglyceride (≥1.70 mmol/L, n,%)	10 (48)

### The Correlations Between Multi-Delay pCASL and CTP in Patients With Ischemic Stroke

**FIGURE 2:**
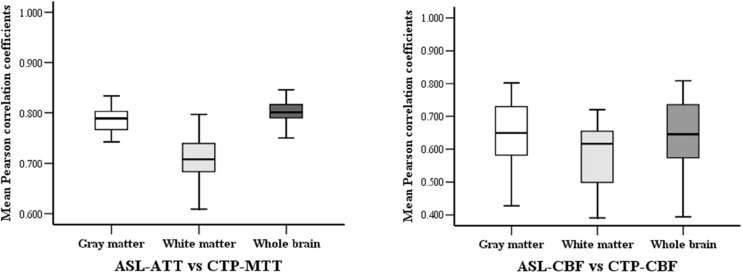
The box-plots of mean Pearson correlation coefficients between the two modalities in gray, white matter and whole brain.

The Pearson correlation coefficients of hemodynamic parameters between two modalities were obtained based on voxel levels in the gray matter, white matter, and whole brain (Table 2; all *P* < 0.005). Moderate to high positive associations between ASL-CBF and CTP-CBF were acquired in the gray matter, white matter and whole brain of the enrolled patients, and the mean Pearson correlation coefficients were 0.647, 0.585, and 0.646, respectively. Highly significant positive correlations between ASL-ATT and CTP-MTT were obtained in the gray matter, white matter and whole brain, and the mean Pearson correlation coefficients were 0.787, 0.707, and 0.799, respectively (Table 3). The box plots showed the same results (Figure 2).

**TABLE 2:** Pearson correlation coefficients between the two modalities in gray, white matter and whole brain of each subject.

	**ASL-ATT vs. CTP-MTT**			**ASL-CBF vs. CTP-CBF**		
**Patient No.**	**Gray matter**	**White matter**	**Whole brain**	**Gray matter**	**White matter**	**Whole brain**
P1	0.781*	0.695*	0.806*	0.604*	0.581*	0.589*
P2	0.803*	0.683*	0.793*	0.779*	0.714*	0.781*
P3	0.743*	0.617*	0.788*	0.730*	0.651*	0.736*
P4	0.757*	0.609*	0.750*	0.736*	0.721*	0.737*
P5	0.767*	0.710*	0.797*	0.600*	0.497*	0.601*
P6	0.783*	0.658*	0.801*	0.802*	0.671*	0.809*
P7	0.788*	0.674*	0.790*	0.569*	0.513*	0.566*
P8	0.792*	0.727*	0.787*	0.662*	0.616*	0.683*
P9	0.787*	0.699*	0.801*	0.612*	0.497*	0.597*
P10	0.825*	0.753*	0.812*	0.705*	0.641*	0.705*
P11	0.801*	0.723*	0.840*	0.718*	0.629*	0.719*
P12	0.754*	0.708*	0.818*	0.748*	0.711*	0.766*
P13	0.757*	0.666*	0.770*	0.475*	0.391*	0.493*
P14	0.742*	0.700*	0.703*	0.427*	0.459*	0.394*
P15	0.814*	0.797*	0.834*	0.574*	0.645*	0.569*
P16	0.799*	0.744*	0.811*	0.650*	0.655*	0.646*
P17	0.789*	0.691*	0.794*	0.522*	0.499*	0.518*
P18	0.834*	0.783*	0.846*	0.582*	0.539*	0.574*
P19	0.822*	0.741*	0.829*	0.637*	0.453*	0.623*
P20	0.790*	0.722*	0.800*	0.765*	0.674*	0.765*
P21	0.807*	0.739*	0.817*	0.697*	0.532*	0.689*

*P<0.005.

**TABLE 3:** Mean Pearson correlation coefficients between the two modalities in gray, white matter and whole brain of 21 patients.

	**Gray matter**	**White matter**	**Whole brain**
ASL-ATT vs. CTP-MTT	0.787 ± 0.026	0.707 ± 0.047	0.799 ± 0.032
ASL-CBF vs. CTP-CBF	0.647 ± 0.102	0.585 ± 0.097	0.646 ± 0.107

### Subgroup Analyses Based on Patient’s Age and Disease Stage

We divided the ages into 30–49, 50–69, and over 70 for a subgroup analysis. The Pearson correlation coefficients exceed 0.5 in each subgroup (Table 4). The results showed a moderate to high positive correlation of different age groups between the two modalities.

**TABLE 4:** Mean Pearson correlation coefficients of different subgroups between the two modalities in gray, white matter and whole brain.

	**ASL-ATT vs. CTP-MTT**			**ASL-CBF vs. CTP-CBF**		
	**Gray matter**	**White matter**	**Whole brain**	**Gray matter**	**White matter**	**Whole brain**
**Age (years)**						
30–49 (*n* = 5)	0.799 ± 0.018	0.732 ± 0.041	0.811 ± 0.020	0.618 ± 0.033	0.558 ± 0.081	0.612 ± 0.044
50–69 (*n* = 9)	0.792 ± 0.030	0.716 ± 0.039	0.802 ± 0.041	0.659 ± 0.126	0.613 ± 0.092	0.657 ± 0.138
>70 (*n* = 7)	0.773 ± 0.023	0.677 ± 0.051	0.787 ± 0.217	0.653 ± 0.107	0.568 ± 0.117	0.655 ± 0.103
**Disease stage**						
Acute (*n* = 5)	0.788 ± 0.027	0.703 ± 0.066	0.799 ± 0.020	0.653 ± 0.106	0.625 ± 0.079	0.657 ± 0.111
Subacute (*n* = 8)	0.784 ± 0.025	0.699 ± 0.046	0.805 ± 0.026	0.701 ± 0.069	0.623 ± 0.081	0.702 ± 0.076
Chronic (*n* = 8)	0.790 ± 0.030	0.716 ± 0.039	0.794 ± 0.043	0.590 ± 0.105	0.523 ± 0.098	0.582 ± 0.109

The comparison of patients at the acute phase, subacute phase, and chronic phase was conducted. The results exhibited a moderate to high correlation in different periods of ischemic stroke patients under the two modalities, indicating the little effect of disease stage.

**FIGURE 3:**
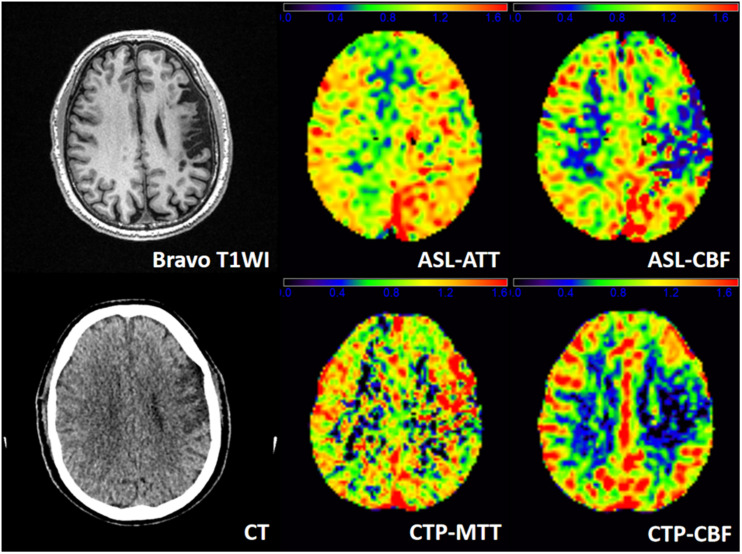
A patient admitted to the hospital with weakness in the right upper limb for 1 day, and he had a history of ischemic stroke last year. T1WI demonstrates an obsolete infarct in the left frontal lobe. ASL-ATT, CTP-MTT, ASL-CBF, and CTP-CBF are normalized images and present consistent results. CTP shows hypoperfused regions in the left frontal lobe. CTP-CBF of the left frontal lobe is lower than the contralateral side, and ASL shows decreased ASL-CBF of the left frontal lobe. CTP-MTT is higher in the infarcted area but not in the surrounding regions, whereas the ASL-ATT is decreased (probably no signal) in the infarcted region and increased in the surrounding regions/territories (fitting with the atrophy patterns).

Figure 3 shows a representative patient of ischemic stroke disease with ASL and CTP images. CTP shows hypoperfused regions in the left frontal lobe. By visual appearance, ASL images present similar perfusion lesion as CTP. However, CTP-MTT is higher in the infarcted area, whereas the ASL-ATT is decreased (probably no signal) in the infarcted region and increased in the surrounding regions/territories (fitting with the atrophy patterns).

**FIGURE 4:**
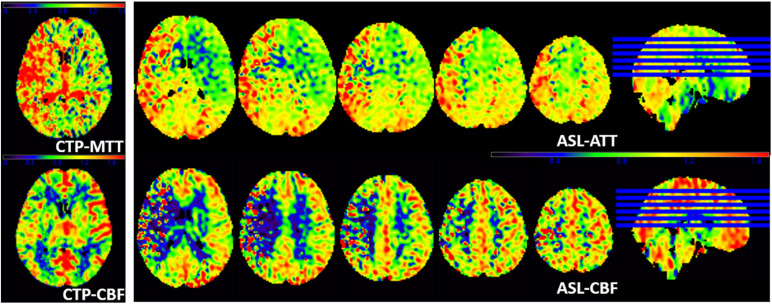
A patient with left limb weakness for 9 days at admission. ASL-ATT, CTP-MTT, ASL-CBF, and CTP-CBF are normalized images and presented consistent results. CTP shows decreased CTP-CBF and increased CTP-MTT in the right frontal, temporal and occipital lobes; ASL shows decreased ASL-CBF and increased ASL-ATT of the right frontal, temporal and occipital lobes compare with the contralateral side. However, ASL-CBF shows focal intravascular signals and low tissue perfusion in the right frontal, temporal and occipital lobes, which may indicate the status of collateral perfusion.

Figure 4 shows another representative case with ischemic stroke disease, and ASL images are in substantial agreement with the results of CTP. Besides, ASL-CBF shows focal intravascular signals and low tissue perfusion in the right frontal, temporal and occipital lobes, which may indicate the status of collateral perfusion.

**FIGURE 5:**
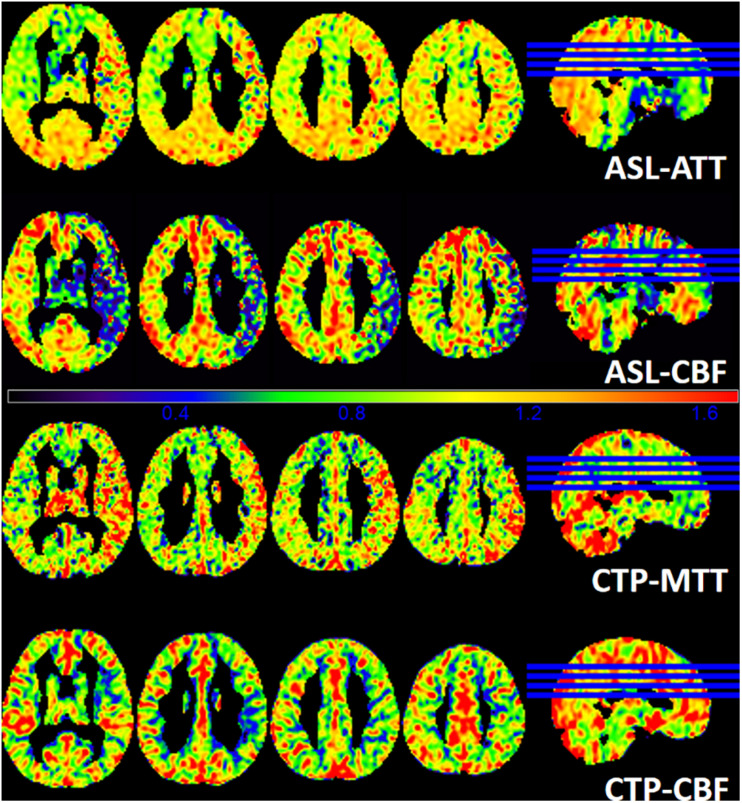
A patient with 6 days of weakness in the right limb at admission. The images demonstrate the gray matter of ASL-ATT, ASL-CBF, CTP-MTT, and CTP-CBF. ASL shows hypoperfusion area of the left frontal, temporal and occipital lobes and basal ganglia. The ASL-CBF of the infarct regions decreases and the ASL-ATT increases compare to the contralateral side. CTP is in concordance with the result of ASL.

ASL-CBF, ASL-ATT, CTP-CBF, and CTP-MTT images were segmented into gray matter maps in Figure 5. ASL shows hypoperfusion area of the left frontal, temporal and occipital lobes and basal ganglia. The ASL-CBF of the infarct regions decreases and the ASL-ATT increases compare to the contralateral side. CTP is in concordance with the result of ASL.

## Discussion

The correlations between pCASL and CT perfusion were analyzed in 21 patients with ischemic stroke in this study. Moderate to high positive associations between ASL-CBF and CTP-CBF were acquired in the gray matter, white matter, and whole brain of the enrolled patients. Wang et al. (2014) also found significant correlations between multi-delay pCASL and CTP in patients with moyamoya disease. CTP scan was initiated after injection of non-ionic iodinated contrast agent. The time concentration curves can be obtained by serially detecting the dynamic CT values of the interest regions (Konstas et al., 2009). In addition, a dynamic assessment of capillary level blood flow through different mathematical models was obtained. ASL adopts magnetically labeled arterial blood water as an endogenous tracer (Wu et al., 2007). When the labeled blood reaches the scanning level, it causes changes in the focal tissue magnetization vector and longitudinal relaxation time. Although the imaging mechanisms of the two modalities are different, they can both reflect blood perfusion of brain. Continuously dynamic scanning is necessary to calculate the CT perfusion parameters; therefore, the required radiation dose increases. Despite the application of low-dose CT scans, radiation damage is still inevitable. Multi-delay pCASL is non-radiative and non-invasive, and can be performed in patients with iodine contrast agent allergies, hyperthyroidism, and renal insufficiency.

The CBF and ATT maps were simultaneously derived using multi-delay pCASL protocol in this study. ATT refers to the time needed for the inverted spins to reach the acquisition region (Chen et al., 2012). The blood flow of ischemic stroke patients is slowed because of stenosis or the collateral pathway of the cerebral arteries, and the arterial transit time is prolonged (Lin et al., 2016). All patients enrolled in this study presented increased ATT in the infarct region, which can be used to detect focal cerebral ischemia. MacIntosh et al. (2010) adopted multi-delay pCASL in patients with acute ischemic stroke. They reported a representative patient with minor lacunar infarcts, and the patient showed obviously high signals on ATT images, which was consistent with our findings. Consequently, we suggest that the measurement of ATT helps to detect subtle ischemic lesions in patients with stroke.

In patients with ischemic stroke, ATT can be prolonged due to intracranial artery stenosis. PLD is defined as the interval time between blood labeling and image acquisition (van der Thiel et al., 2018). When the PLD is closer to the ATT, the CBF value is closer to the cerebral blood flow under physiological conditions. If the PLD cannot be adjusted in a timely manner, some of the labeled blood fails to reach the acquisition level due to delayed arrival, which may lead to underestimation of the perfusion (Ferré et al., 2013). The International Society for Magnetic Resonance in Medicine and the European Consortium ASL in Dementia recommends that the PLD of adults with cerebrovascular disease should be set to 2 s (Alsop et al., 2015). When patients have severe cerebrovascular stenosis, the ATT will be significantly prolonged because of flow deficits. Therefore, 2 s may still result in underestimation of the CBF. In recent years, a single PLD was generally employed in most ASL studies, and the delay time was usually between 1.5 and 2 s (Chen et al., 2018; Jezzard et al., 2018). In our study, a total of 6 PLDs between 1,000 and 3,500 ms were adopted to improve the accuracy of cerebral perfusion quantitative measurement in ischemic patients. However, the multi-delay pCASL also has some limitations. As the delay time increases, the scanning time will also increase, as will the possibility of motion artifacts. Therefore, this imaging protocol is suitable only for patients in a stable condition and with high levels of cooperation.

In our study, we found a patient that presented focal intravascular signals and low tissue perfusion in the infarct regions. Recent clinical evaluations of ASL in cerebrovascular disease have shown that focal intravascular signals and the delayed arterial transmission of tissue hypoperfusion may indicate the status of collateral perfusion (Chen et al., 2009; Zaharchuk, 2011; Lou et al., 2017). Therefore, we speculate that another potential ability of multi-delay pCASL could be evaluating collateral flow through dynamic perfusion images.

Evaluating cerebral perfusion plays an important role in determining treatment options for patients with ischemic stroke. Some patients also need regular follow-up brain perfusion examinations to assess the treatment efficacy and make timely adjustments. Up to present, few studies on multi-delay ASL have been reported (Wang et al., 2013). Compared with traditional single PLD ASL, multi-delay pCASL allows for non-invasive perfusion imaging and can provide more relevant clinical information. Besides, we have increased the comparison of patients at the acute phase, subacute phase, and chronic phase. The results showed a moderate to high correlation in different periods of ischemic stroke patients between ASL and CTP, indicating the little effect of disease stage. In addition, a moderate to high correlation of different age groups under the two modalities was obtained. Therefore, we speculated multi-pCASL could be used as an alternative imaging method for CTP.

In this study, a total of 3 female patients were included, far fewer than men. However, we calculated the correlation coefficients of each subject under the two modalities. The results of the three ischemic female patients were basically consistent with the overall trend. Multi-delay pCASL is non-radiative and non-invasive, and can be combined with other MRI sequences, such as anatomical imaging, vascular imaging, and diffusion weighted imaging; thus, more imaging information can be obtained during one examination.

There are some limitations in our study. First, only 21 patients were enrolled in this study, but presented a relatively representative cohort of patients with ischemic stroke. Second, gender bias should be excluded, as the female patients were far fewer than male patients. Despite we calculated the correlation coefficients of each subject under the two modalities, we will continue to include more female patients to verify this conclusion.

## Conclusion

In conclusion, the present study showed moderate to high significant correlations between perfusion parameters from multi-delay pCASL and CTP imaging in patients with ischemic stroke. Due to the clinical feasibility and utility of multi-delay pCASL, it could potentially be used as part of a standard neuroimaging protocol for the management of ischemic stroke disease.

## Data Availability Statement

The raw data supporting the conclusions of this article will be made available by the authors, without undue reservation.

## Ethics Statement

The studies involving human participants were reviewed and approved by the institutional review board of the First Affiliated Hospital of Jinan University. The patients/participants provided their written informed consent to participate in this study.

## Author Contributions

CS and LL designed the study. XX was a major contributor in writing the manuscript. ZT and MF contributed to collect the imaging and clinical data. XX, ZT, and MF performed data analysis and interpretation. MM, WF, JL, and ZX revised it critically for important content. All authors have read and approved the manuscript.

## Conflict of Interest

The authors declare that the research was conducted in the absence of any commercial or financial relationships that could be construed as a potential conflict of interest.

## Publisher’s Note

All claims expressed in this article are solely those of the authors and do not necessarily represent those of their affiliated organizations, or those of the publisher, the editors and the reviewers. Any product that may be evaluated in this article, or claim that may be made by its manufacturer, is not guaranteed or endorsed by the publisher.

## Footnotes

1
http://restfmri.net/forum/DPABI

2
http://www.fil.ion.ucl.ac.uk/spm/
